# Pro-Cognitive Effect of Royal Jelly Is Linked with Increased Burst Activity of Mesocorticolimbic Dopaminergic Neurons

**DOI:** 10.3390/nu17223593

**Published:** 2025-11-17

**Authors:** Katarína Bíliková, Daniela Jezova, Daniil Grinchii, Henrieta Oravcová, Tatiana Krištof Kraková, Ruslan Paliokha, Hande Özbaşak, Matej Račický, Eliyahu Dremencov

**Affiliations:** 1 Institute of Molecular Physiology and Genetics, Centre for Biosciences, Slovak Academy of Sciences, 845 05 Bratislava, Slovakia; k.bilikova@savba.sk (K.B.); daniil.grinchii@savba.sk (D.G.); t.krakova@savba.sk (T.K.K.); ruslan.paliokha@savba.sk (R.P.); hande.ozbasak@savba.sk (H.Ö.); matej.racicky@savba.sk (M.R.); 2 Institute of Experimental Endocrinology, Biomedical Research Center, Slovak Academy of Sciences, 845 05 Bratislava, Slovakia; daniela.jezova@savba.sk (D.J.); henriet.or@gmail.com (H.O.)

**Keywords:** novel object recognition, dorsal raphe nucleus (DRN), serotonin (5-HT), locus coeruleus (LC), noradrenaline, ventral tegmental area (VTA), dopamine

## Abstract

**Background:** Royal jelly is a protein-rich honeybee secretion that is used in the nutrition of larvae and adult queens. Previous studies have reported that royal jelly had induced pro-cognitive, anxiolytic, and antidepressant-like effects in laboratory rats. Since serotonin (5-HT), noradrenaline, and dopamine play an important role in the control of several mental functions, changes in the excitability of monoaminergic neurons may be involved in the mechanisms of the behavioral and neurochemical effects of royal jelly. The present study aimed to test this hypothesis. **Methods:** Adult male Wistar rats were treated with royal jelly for two weeks. Thereafter, their cognitive performance was evaluated using the novel object recognition (NOR) test. The excitability of monoaminergic neurons was assessed using in vivo single-unit extracellular electrophysiology. **Results:** We found that rats treated with royal jelly had a higher recognition index in the NOR test and a higher burst activity of dopaminergic neurons of the ventral tegmental area (VTA) compared to the vehicle-treated controls. The firing activities of 5-HT neurons of the dorsal raphe nucleus (DRN) and the noradrenergic neurons of the locus coeruleus (LC) were not altered. **Conclusions:** We conclude that the pro-cognitive effect of royal jelly is mediated, at least in part, by mechanisms involving the excitability of mesolimbic dopaminergic neurons. The present findings encourage further research towards the improvement of the safety and efficacy of currently available therapies for cognitive dysfunction.

## 1. Introduction

Royal jelly is a yellowish, creamy, acidic secretion of the hypopharyngeal and mandibular glands of nurse bees that serves as food for honeybee larvae and throughout the entire life of the honeybee queen. This precious substance is rich in biologically active compounds that make one it of the most valuable natural products that has been mainly used in traditional medicine. The major parts of royal jelly are water (60–70% *w*/*w*), proteins (9–18% *w*/*w*), sugars, (e.g., glucose, fructose, and sucrose, 7–18% *w*/*w*), and lipids and fatty acids (e.g., 10-Hydroxy-2-decenoic, stearic, and 3,10-dihydroxydecanedioic acids, 3–8% *w*/*w*), and the minor components include minerals (K, Ca, Ca, Mg, Zn, Fe, and Cu, 250–1250 mg/100 g), amino acids (e.g., tyrosine, 8–60 g/100 g), vitamins (e.g., B1, B2, B3, B6, and folic acid, 5–25 mg/100 g), hormones (e.g., testosterone, prolactin, and estradiol), nucleotides (e.g., adenosine and its monophosphate), enzymes, polyphenols, and flavonoids [1]. The key proteins of royal jelly are major royal jelly proteins (MRJPs), which belong to a large protein family of nine members with molecular masses in the range of 49–87 kDa [2,3]. The most abundant royal jelly protein, MRJP1 (also called apalbumin1) [4], occupies an exclusive position because it is simultaneously synthesized in the honeybee brain [5] as well as in the hypopharyngeal glands of adult honeybees [6,7]. It was found that MRJP1 plays an important role in larval development into a queen rather than into a working bee [8] and is also responsible for many healing properties of royal jelly. Queens and working bees are genetically similar, and their phenotypical difference is determined by epigenetic mechanisms, such a methylation of the specific areas of the genome. MRJPs thus act as epigenetic modulators [9].

Royal jelly has been demonstrated to possess a broad range of functional properties, such as anti-inflammatory, immunomodulatory, neuroprotective, vasodilatory, antidiabetic and hypotensive activities, anti-bacterial, anti-allergic, and anti-osteoporotic and antioxidant effects, anti-hypercholesterolaemic activity, and antitumor properties [10,11,12,13,14,15]. Ghorbanpour and colleagues [16] reported that royal jelly induced pro-cognitive, anxiolytic, and antidepressant-like effects in laboratory rats exposed to stressors. Thus, royal jelly increased the novel object recognition scores and decreased the time spent in the open arms of the elevated plus maze apparatus. In the same study, it was reported that treatment with royal jelly attenuated the stress-induced increase in circulating glucocorticoid concentrations and decrease in levels of brain derived neurotrophic factor (BDNF; a key neuroprotector and modulator of neuroplasticity) [17].

It is well established that the corticolimbic serotonergic (5-HT), noradrenergic, and dopaminergic circuits play a fundamental role in memory, cognition, and mood regulation [18]. These systems are also the key modulators of BDNF expression; monoamine–BDNF interaction is involved in the pathophysiology and treatment of depression, anxiety, and cognitive disorders [17]. It is therefore likely that the pro-cognitive effects of the royal jelly and its specific component(s) are mediated, at least in part, via mechanism(s) involving the alteration of the excitability of monoaminergic neurons. The primary goal of this study was to test this hypothesis.

Substantial portions of 5-HT, noradrenergic, and dopaminergic neurons projecting to the corticolimbic areas are located in the dorsal raphe nucleus (DRN), locus coeruleus (LC), and ventral tegmental area (VTA), respectively. The generation of action potentials or spikes in the cell bodies of monoaminergic neurons within these three brain nuclei is likely to determine limbic monoaminergic transmission. With respect to monoaminergic neurotransmission, next to the overall firing rate, the mode of the spike generation is of crucial importance. The burst-like or phasic mode of firing of monoaminergic neurons (e.g., generation of the cluster of spikes characterized by millisecond-range inter-spike interval, followed by a second-range period of silence) is more efficient in terms of neurotransmission than the tonic mode of activity, when the same number of action potentials are generated in a single-spike mode [19]. To address these aspects of monoaminergic neurotransmission, we assessed the effect of royal jelly on the firing rate, as well as on the burst activity of the neurons.

## 2. Materials and Methods

### 2.1. Royal Jelly Preparation

Fresh honeybee royal jelly was obtained from Japan Royal Jelly, Co., Ltd., Tokyo, Japan. Royal jelly suspension at a concentration of 200 mg/mL was prepared by mixing 10 g of fresh royal jelly in 25 mL of Tris-buffered saline, pH 7.5 (TBS), and filling up to 50 mL of total volume with TBS buffer. Suspension of royal jelly was frozen in 500 µL aliquots at −80 °C before experimental use.

### 2.2. Animals

**Figure 1:**
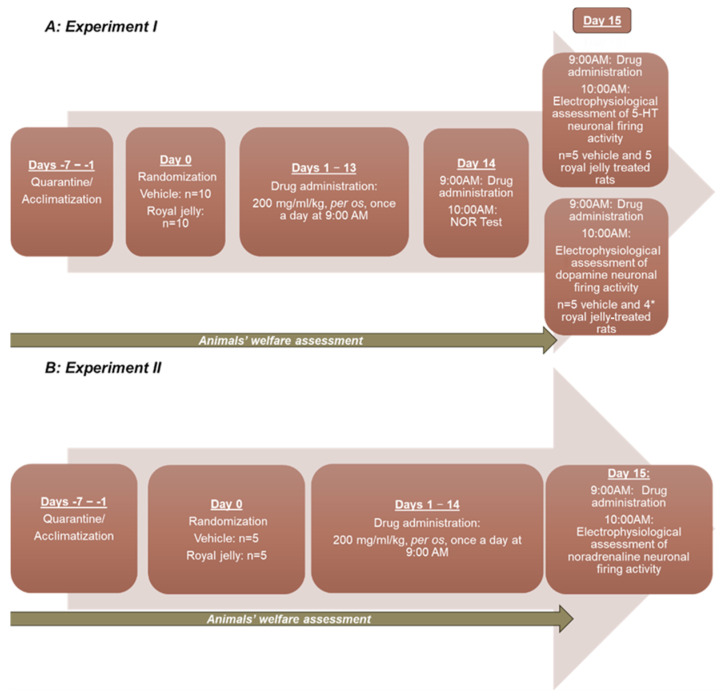
Experimental design; * one royal jelly-treated animal died from anesthesia before the beginning of electrophysiological recordings.

Adult male Wistar rats, weighing 250–350 g, were ordered from the Animal Breeding facility of the Institute of Experimental Pharmacology and Toxicology, Centre for Experimental Medicine, Slovak Academy of Sciences (Dobra Voda, Slovakia). Animals were housed under standard laboratory conditions (temperature 22 ± 2 °C, humidity: 55%) with a 12 h light/12 h dark cycle (lights on at 6 a.m.). Pelleted food and tap water were available ad libitum. The experiments were planned in accordance with the ‘3Rs’ strategy (Replacement, Reduction, and Refinement). All experimental procedures were approved by the Animal Health and Animal Welfare Division of the State Veterinary and Food Administration of the Slovak Republic (Permit number Ro 2019/2022-220) and conformed to the Directive 2010/63/EU of the European Parliament and of the Council on the Protection of Animals Used for Scientific Purposes. The detailed experimental design is illustrated in Figure 1:

### 2.3. Repeated Administration of the Royal Jelly

After a seven-day quarantine period, designated to exclude the presence of pathogens and to allow the animals to acclimatize to the animal facility, the animals were randomly divided into two groups: royal jelly and vehicle (TBS). The number of animals per group (n = 10/group in the novel object recognition: NOR experiments with the subsequent assessment of firing activity of 5-HT and dopaminergic neurons and n = 5/group for the assessment of the firing activity of noradrenergic neurons) was determined by power analysis based on the results of our previous studies [20,21,22]. The royal jelly TBS solution was administered per os, at a dose of 200 mg/mL/kg, for fifteen consecutive days, once daily at 9 a.m.; control animals were administered vehicle. The dose and the route of administration were set in accordance with a previous study [16].

### 2.4. NOR Test

The behavioral test was performed one hour following the royal jelly or vehicle administration on the 14th day of chronic treatment. The NOR test was performed as previously described [20,21]. In short, each rat was allowed to explore two identical objects placed in a square-shaped arena (50 × 50 cm) for five minutes. Thereafter, the rat was put into a separate cage for three minutes while one of the familiar objects was replaced by a new one. Next exposure to the arena lasted five minutes, and the exploration time of each object was measured using a computer program. The results were expressed in the form of a recognition index that was calculated as the ratio of the time spent on exploration of the new object and the sum of the time spent on exploration of both the new and the old object × 100%.

### 2.5. In Vivo Electrophysiology

In vivo electrophysiological assessments were performed one hour following the royal jelly or vehicle administration on the 15th day, as previously described [22]. Animals were anesthetized by chloral hydrate (400 mg/kg, i.p.) and mounted in the stereotaxic frame (David Kopf Instruments, Tujunga, CA). Body temperature was maintained between 36 and 37 °C with a heating pad (Gaymor Instruments, Orchard Park, NY, USA). The scalp was opened, and a 3 mm hole was drilled in the skull for insertion of electrodes.

Glass pipettes were pulled with a DMZ-Universal Puller (Zeitz-Instruments GmbH, Martinsried, Germany) to a fine tip approximately 1 μm in diameter and filled with 2 M NaCl solution. Electrode impedance ranged from 4 to 6 MΩ. The pipettes were inserted into the DRN (7.8–8.3 mm posterior to bregma and 4.5–7.0 mm ventral to brain surface), LC (8.0–8.3 mm posterior to bregma, 1.2–1.4 mm lateral to the midline, and 5.5–7.5 mm ventral to the brain surface), or VTA (4.5–5.5 mm posterior to bregma, 0.6–0.8 mm lateral to the midline, and 7.0–8.5 mm ventral to the brain surface) [23] by a hydraulic micro-positioner (David Kopf Instruments, Tujunga, CA, USA). The action potentials generated by monoamine-secreting neurons were recorded using the AD Instruments Extracellular Recording System (Dunedin, New Zealand). After completion of the electrophysiological recordings, the animals were euthanized by overdose of chloralhydrate.

The 5-HT neurons were identified by bi- or tri-phasic action potentials with a rising phase of long duration (0.8–1.2 ms) and regular firing rate of 0.5–5.0 Hz [22]. Noradrenergic LC neurons were recognized by action potentials with a long-duration rising phase (0.8–1.2 ms), regular firing rate of 0.5–5.0 Hz, and a characteristic burst discharge in response to nociceptive pinch of the contralateral hind paw [24]. Dopaminergic neurons were recognized by tri-phasic action potentials lasting between 3 and 5 ms with a rising phase lasting over 1.1 ms, inflection or “notch” during the rising phase, marked negative deflection, irregular firing rate of 0.5–10 Hz, and mixed single-spike and burst firing with characteristic decrease of the action potentials amplitude within the bursts [25].

### 2.6. Electrophysiological Data Analysis

Action potentials generated by 5-HT, noradrenergic, and dopaminergic neurons were detected using the spike sorting algorithm, with the version 6.02 of Spike2 software (Cambridge Electronic Design, Cambridge, UK). The neuronal firing rate and burst activity characteristics were calculated using the burstiDAtor software (www.github.com/nno/burstidator; accessed on 12 November 2025). The onset of a burst was signified by the occurrence of two spikes with ISI < 0.08 s for noradrenaline and dopamine neurons, and ISI < 0.01 s for 5-HT neurons. The termination of a burst was defined as an ISI > 0.16 s for noradrenaline and dopamine neurons [26,27] and ISI > 0.010s for 5-HT neurons [28]. The following characteristics of the neuronal firing activity were assessed: mean number of the spontaneously active neurons per electrode track, mean firing rate (Hz), and mean frequency of the bursts (Hz), percent (%) of spikes occurring in the bursts, and mean number of spikes in burst.

### 2.7. Statistical Analysis

Statistical assessments were performed using SigmaPlot 12.5 software (Systat Software Inc, Chicago, IL, USA). Single animals were considered experimental units in the NOR experiments. Single neurons were considered experimental units in electrophysiology experiments. Data was shown as mean ± standard error or mean (SEM). Two-tailed Student’s *t*-test, preceded by F-test assessment of the variance, was used to determine the effect of royal jelly on the NOR scores and excitability of monoaminergic neurons. The probability of *p* ≤ 0.05 was considered significant.

## 3. Results

### 3.1. Repeated Treatment with Royal Jelly Improved Cognitive Performance in the NOR Test

**Figure 2:**
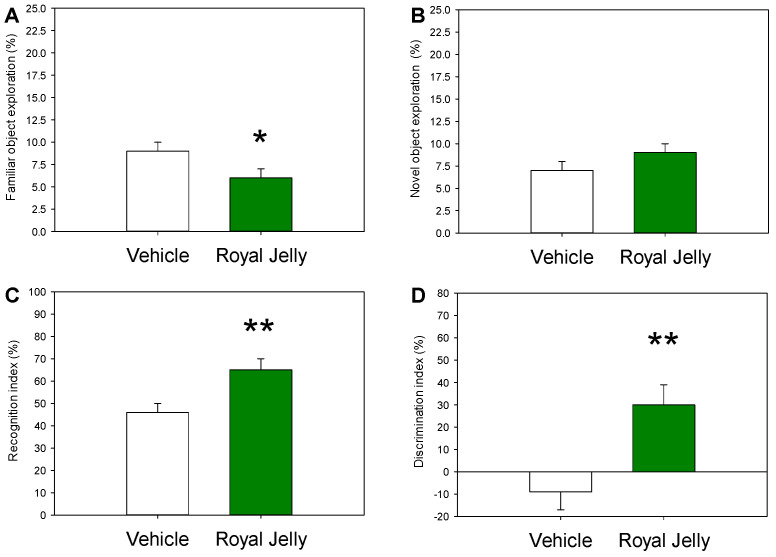
Effect of the chronic treatment with royal jelly on familiar (**A**) and novel (**B**) object exploration and on recognition (**C**) and discrimination (**D**) indexes in the NOR test; * *p* < 0.05 and ** *p* < 0.01 in comparison with vehicle, two-tailed Student’s *t*-test (n = 10 rats in each group).

Figure 2 illustrates the effect of two-week administration of royal jelly on the rats’ performance during the NOR test.

Treatment with royal jelly had a positive impact on all four parameters evaluated in the NOR test. Royal jelly-treated rats spent significantly (*p* = 0.04) less time in exploration of the familiar object and numerically more time with the novel object exploration. Most importantly, values of the recognition index found in royal jelly-treated animals were significantly higher than those in vehicle-treated controls (*p* = 0.007). Similarly, the royal jelly-treated rats displayed higher discrimination index values compared to vehicle-treated controls (*p* = 0.007). No adverse effects of royal jelly, such as weight loss or reduction in the water or food consumption or home-cage activity were observed, therefore, no endpoints were applied, and no animals were excluded from the study.

### 3.2. Royal Jelly Did Not Alter the Excitability of 5-HT Neurons of the DRN

**Figure 3:**
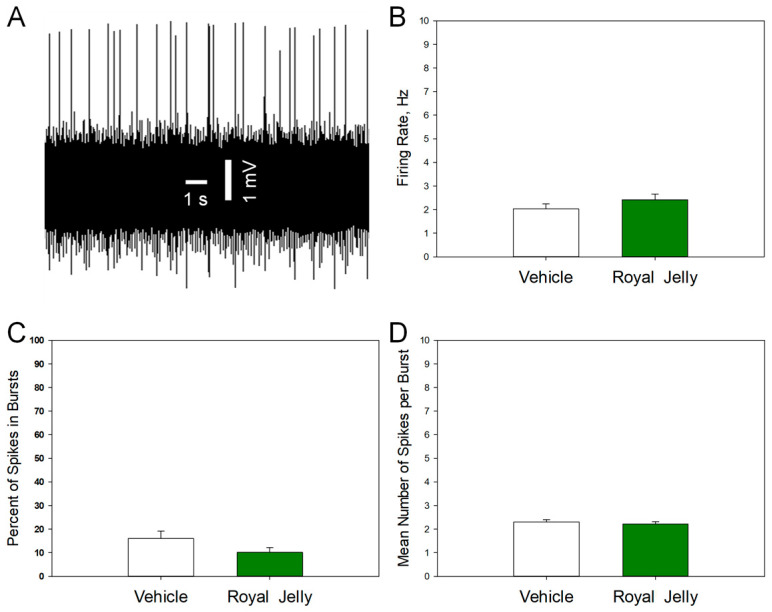
Characteristics of the excitability of 5-HT neurons of the DRN in the vehicle (n = 48 neurons from 5 rats)- and royal jelly (n = 48 neurons from 5 rats)-treated rats; (**A**): representative recording from a DRN 5-HT neuron; (**B**): mean spontaneous firing rate; (**C**): percent of spikes in bursts; (**D**): mean number of spikes in burst.

Figure 3 shows a representative recording from DRN 5-HT neurons (A) and illustrates the mean spontaneous firing rate (B), percentage of spikes occurring in bursts (C), and mean number of spikes per burst (D) of 5-HT neurons in the vehicle- and royal jelly-treated rats. No differences between the groups were observed. Other characteristics of neuronal firing activity, such as the mean number of the spontaneously active neurons per electrode track and mean frequency of the bursts, not shown in the illustration, were also not affected by chronic royal jelly treatment.

### 3.3. Royal Jelly Did Not Alter the Excitability of Noradrenergic Neurons of the LC

**Figure 4:**
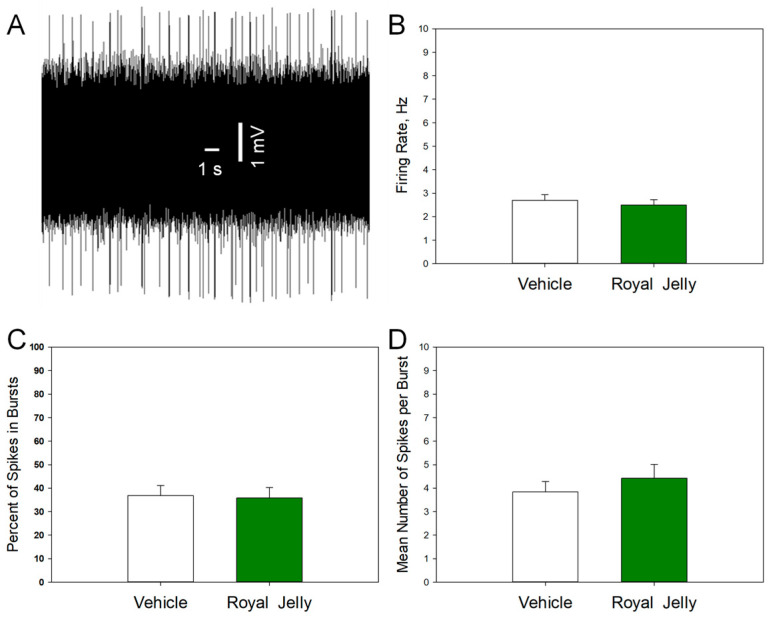
Characteristics of the excitability of noradrenergic neurons of the LC in the vehicle (n = 41 neurons from 5 rats)- and royal jelly (n = 41 neurons from 5 rats)-treated rats; (**A**): representative recording from a DRN 5-HT neuron; (**B**): mean spontaneous firing rate; (**C**): percent of spikes in bursts; (**D**): mean number of spikes in burst.

Figure 4 shows a representative recording from an LC noradrenergic neuron (A) and illustrates the mean spontaneous firing rate (B), percentage of spikes occurring in bursts (C), and mean number of spikes per burst (D) of noradrenergic neurons in the vehicle- and royal jelly-treated rats. No differences between the groups were observed. Other characteristics of neuronal firing activity, such as the mean number of the spontaneously active neurons per electrode track and mean frequency of the bursts, not shown in the illustration, were also not affected by chronic royal jelly.

### 3.4. Royal Jelly Enhanced the Burst Firing of Dopamine Neurons of the VTA

**Figure 5:**
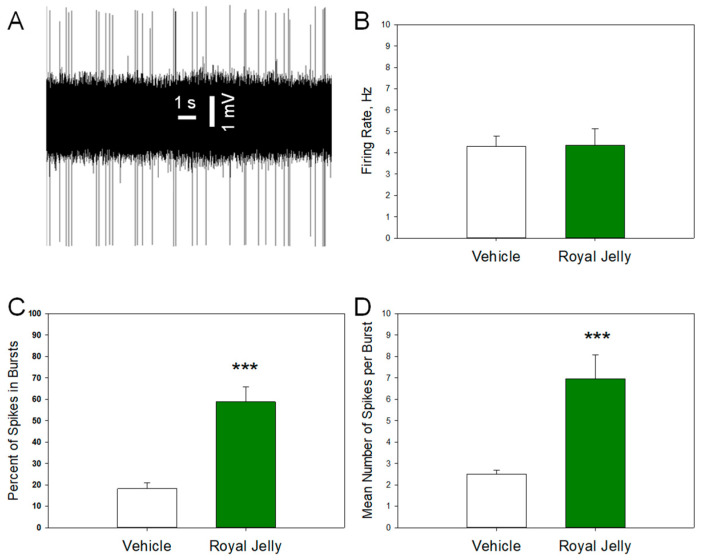
Characteristics of the excitability of dopaminergic neurons of the VTA in the vehicle (n = 44 neurons from 5 rats)- and royal jelly (n = 18 neurons from 4 rats)-treated rats; (**A**): representative recording from a DRN 5-HT neuron; (**B**): mean spontaneous firing rate; (**C**): percent of spikes in bursts; (**D**): mean number of spikes in burst; *** *p* < 0.001, two-tailed Student’s *t*-test.

Figure 5 shows a representative recording from a DRN 5-HT neuron (A) and illustrates the mean spontaneous firing rate (B), percentage of spikes occurring in bursts (C), and mean number of spikes per burst (D) in the vehicle- and royal jelly-treated rats. No differences in the mean spontaneous firing rate between the groups were observed.

The percentage of neurons occurring in the bursts (*p* = 0.00001) and mean number of spikes in bursts (*p* = 0.001, two-tailed Student’s *t*-test) in the royal jelly-treated rats were, however, significantly higher compared to the vehicle-treated controls. Other characteristics of 5-HT neuronal firing activity, such as the mean number of the spontaneously active neurons per electrode track and mean frequency of the bursts, not shown in the illustration, were not affected by chronic royal jelly treatment.

## 4. Discussion

The main findings of the present study are that chronic treatment of rats with royal jelly led to improved cognitive performance and increased burst firing of dopaminergic neurons. The mean firing rate of 5-HT, noradrenergic, and dopaminergic neurons, as well as burst firing of noradrenergic and 5-HT neurons, were not affected by the treatment with royal jelly.

We found that the rats treated with royal jelly had a higher recognition index in the NOR test than the vehicle-treated controls. This finding is in strong agreement with the data previously observed and published by Ghorbanpour and colleagues [16]. Similar to the present study, these authors used novel object recognition as a behavioral test to assess cognitive performance. In contrast to the present study, they initially exposed the rats to two stressors to evoke stress-induced cognitive impairment. Thus, the results obtained in the present study suggest that treatment with royal jelly results in pro-cognitive effects even in the absence of induction of cognitive function impairments.

Our finding on the pro-cognitive effect of royal jelly is also consistent with the report of Pyrzanowska and co-authors on the spatial memory-improving effect of long-term administration of this honeybee product in aged rats [29]. Royal jelly is a natural compound whose exact composition varies depending on the honeybee species, nectar source, climatic conditions, season, and other environmental factors [30]. Therefore, the fact that results obtained in one laboratory using one batch of royal jelly can be reproduced in another laboratory using a different batch is of crucial importance. Such replication of the results suggests that the pro-cognitive behavioral effects of royal jelly are likely attributable to one or more of its core chemical components—such as MRJPs—which are consistently present across different batches, rather than depending on a specific source of the product. It can be noted that a pro-cognitive effect in rodents was observed following administration of another natural honeybee product, propolis [31].

The electrophysiological measurements in the present study failed to reveal any effects of repeated royal jelly administration on the excitability of 5-HT neurons of the DRN and noradrenergic neurons of the LC. It may be speculated that the pro-cognitive, anxiolytic, and antidepressant-like effects of royal jelly reported in previous studies [16,29], are not dominantly induced via mechanisms involving the brain 5-HT and/or noradrenergic systems. Alternatively, royal jelly still can alter 5-HT and noradrenergic neurons by a mechanism not involving action potential generation in the cells releasing these neurotransmitters.

An important finding of the present study is the enhancement of burst firing of dopamine neurons induced by repeated treatment with royal jelly. Since the burst mode of firing of dopamine neurons is associated with higher efficiency of the nerve terminal transmitter release [19], the administration of royal jelly is likely to induce an augmentation of mesocorticolimbic dopamine neurotransmission. To the best of our knowledge, our study is the first to examine the effect of royal jelly on dopamine neurotransmission in a vertebrate species. It is known however that intake of tyrosine in royal jelly can increase dopamine concentration in the honeybee neural system [32]. Furthermore, pro-dopaminergic effect of the royal jelly is responsible, at least in part, for the pigmentation pattern creation in honeybee queens [33] and for the transition from normal to reproductive workers in queen-less honeybee colonies [34].

Among others, mesocorticolimbic dopaminergic neurotransmission is fundamental in cognitive functions, such as memory and attention. Particularly, the linkage between the behavior of rats in the NOR test and the firing activity of the VTA dopaminergic neurons was demonstrated in previous studies from our laboratory [21], as well as from others [35,36]. It is thus likely the that pro-cognitive, memory-improving effects of royal jelly, observed in previous studies [16,29], as well as in the present work, are based on a mechanism involving the VTA dopaminergic system. In a different animal model, it was shown that experimental improvement of a spatial learning memory deficit was associated with increased dopamine synthesis and enhanced expression of BDNF [37]. It is thus possible that BDNF contributes to the suggested dopamine-mediated pro-cognitive effect of royal jelly. Indeed, the ability of royal jelly to stimulate BDNF expression in the vertebrate brain has been previously reported [17].

The primary limitation of the present study is the lack of identification of the exact biological molecule(s) responsible for the pro-dopaminergic and pro-cognitive effect of the royal jelly. Royal jelly is rich in the amino acid tyrosine, a precursor or dopamine. As mentioned above, royal jelly, as well as tyrosine, increased dopamine levels in the honeybee brain [32,34]. It is thus possible that royal jelly increases dopamine concentrations in the vertebrate brain as well. An acute increase in extracellular dopamine levels, however, may lead to the activation of dopamine-2 (D_2_) autoreceptors, and inhibition rather than stimulation of the central dopaminergic neurons. Nevertheless, chronic intake of compounds decreasing the excitability of dopaminergic neurons, such as trace amine associate receptor 1 (TAAR1) agonist RO5256390 [38] or delta opioid receptor agonist SNC80 [39], led to an excitation of mesolimbic dopaminergic neurons. Desensitization of the D_2_ autoreceptors has been proposed as a mechanism responsible for the switch from inhibition by an acute intake and excitation by chronic intake of RO5256390 or SNC80 [38,39]. It is thus possible that chronic royal jelly intake leads to desensitization of the central D_2_ autoreceptors as well.

Alternatively, MRJP(s), as epigenetic modulators [9], might modulate expression of the genes coding for proteins regulating dopamine transmission, such as D_2_ autoreceptor, dopamine transporter (DAT), and/or enzymes involved in dopamine synthesis and metabolism, such as tyrosine hydroxylase. There is some evidence that royal jelly may lead to methylation of the genes ALDH, ALDH7A1, and OGDH, playing an important role in tyrosine metabolism and dopaminergic signaling in honeybees [33].

**Figure 6:**
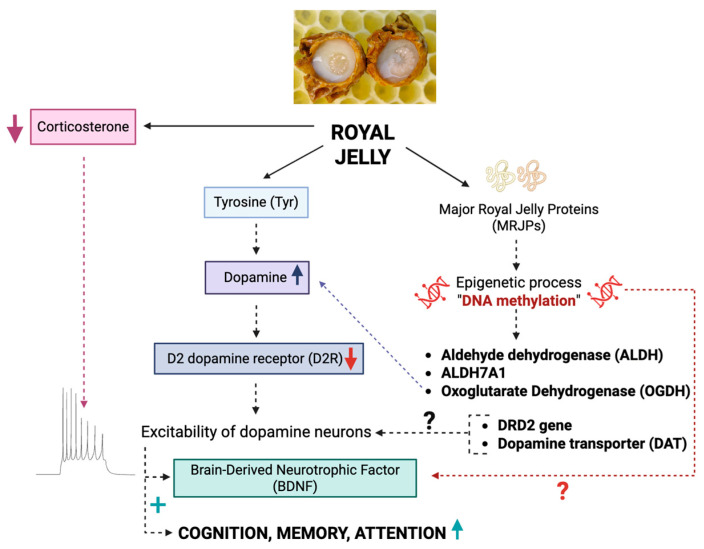
Putative mechanisms underlying the dopamine-mediated pro-cognitive effect of royal jelly.↑, increase; ↓, decrease; +, stimulatory effect.

Finally, since corticosterone has a suppressive effect on dopaminergic neurons [40], and since royal jelly decreases corticosterone levels in rats [41], glucocorticoid signaling might be responsible, at least in part, for the pro-dopaminergic effect of royal jelly. These hypotheses should be tested in future studies. Putative mechanisms underlying dopamine-mediated pro-cognitive effects of royal jelly are illustrated in Figure 6. Another limitation is that the researchers were not blinded to the group assignment of the animals.

## 5. Conclusions

The results of the present experiments confirmed our working hypothesis, showing that the excitability of dopaminergic neurons, though not that of serotoninergic and noradrenergic neurons, is associated with pro-cognitive effects induced by a two-week treatment with royal jelly in male middle-aged rats. The present results encourage further research towards the improvement of the safety and efficacy of currently available therapies for cognitive dysfunction.

## Data Availability

The raw data supporting the conclusions of this article will be made available by the authors on request.

## Abbreviations

The following abbreviations are used in this manuscript:

5-HT	Serotonin
BDNF	Brain derived neurotrophic factor
DRN	Dorsal raphe nucleus
ISI	Interspike interval
LC	Locus coeruleus
MRJP	Major royal jelly protein
NOR	Novel object recognition
TBS	Tris-buffered saline
VTA	Ventral tegmental area
